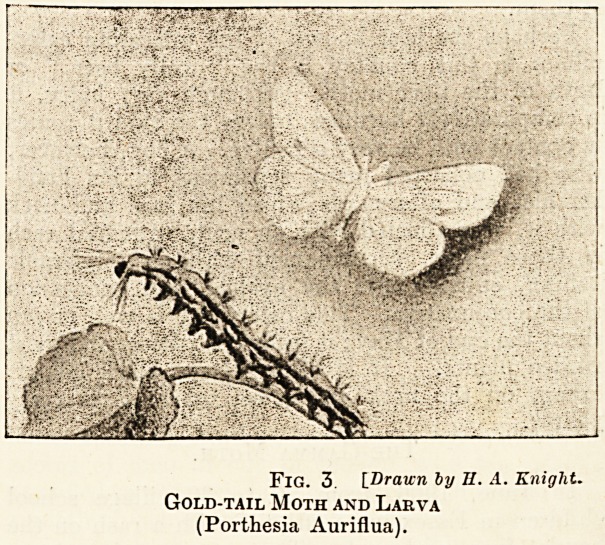# Venomous Caterpillars

**Published:** 1908-08-22

**Authors:** Edward Knight


### SPECIAL ARTICLE.

### VENOMOUS CATERPILLARS.

By EDWARD KNIGHT, L.R.C.P., L.R.C.S., Ed.

In most text-books on skin diseases we find among various causes of nettle rash "certain hairy caterpillars," but not always a definite description of them.

Perhaps the best known of the urticating caterpillars in this country is the "wolly bear," or larva of the tiger moth. Although very hairy, it is comparatively harmless unless the skin it comes in contact with be very sensitive. A boy, however, who had collected many specimens in his handkerchief and afterwards wiped his face and neck with it, was affected with an acute urticarial rash in those parts, accompanied with an intolerable itching, effusion into the eyelids, and congestion of the conjunctiva. This caterpillar is well grown about the end of May; it feeds on nettles, and most garden plants. It is of a velvety black colour, except on the neck and sides, where it is rusty yellow.

### The Gamma Moth.

In June, 1906, from 20 to 30 village school children in Essex were affected with a rash on the hands, face, and neck. There was much redness and irritation, and some swelling around the eyes. In every instance it was found that the child attacked had been handling the caterpillar of the gamma moth, which was feeding in large numbers on the hawthorn hedges. Its colour is generally green, and it has fine scattered hairs upon it; it is found from spring to autumn. The head is brownish green. On the back are four yellowish or whitish stripes, and above the legs is a yellow stripe; the spiracles are dark green. The moth is most often light or dark grey, and gets its name

Fig. 1. [*Drawn by H. A. Knight.*

Gamma Moth and Larva

(Ilusia Gamma).

from having a silvery or golden mark on the fore-wings that is thought to be like the greek letter gamma ($\gamma$).

During the following July, there was a remarkable caterpillar plague in London. Many of the trees in Hyde Park were almost stripped of their leaves by the ravages of the larvæ of the vapourer moth. They fell from the trees and swarmed upon the ground and climbed up the legs of each chair and bench; no person could sit on a seat without getting them on him. Their hairs irritate a delicate skin, and if transferred from the hands to the eyes cause conjunctivitis. Caterpillar-ophthalmia has been made the subject of a treatise by Meixner. The larva is variable in colour and is recognised by having two tufts of black hairs on the second segment directed forwards, and a single tuft on the last but one directed backwards. Also it has a brush-like tuft of yellow hairs on the fifth, sixth, seventh, and eighth segments. The male moth is chestnut brown, and has a white crescentic spot on each forewing. It flies with a vapouring kind of motion, hence its name. The female has only rudiments of wings and cannot fly.

FIG. 2. [*Drawn by H. A. Knight.*
VAPOURER MOTH
(Orgia Antiqua. Male and Female and Larva).

### THE GOLD-TAIL MOTH.

The Rev. J. G. Wood found his face and the backs of his hands on three occasions, after dissecting the larvæ of the gold-tail moth, "swollen into hard knots, as if moderate-sized potatoes had been inserted under the skin." These caterpillars are bright scarlet and black, and have tufts of hairs on their bodies like camel-hair brushes. The moth is covered with a downy white plumage, and has at the end of its tail a tuft of golden hair. Another entomologist states that in his experience the caterpillar of the brown-tail moth causes a more troublesome eruption. This moth is a satiny-white, the hinder part of its body ending in a tuft of brown hairs in the males, and rust-coloured in the females. The caterpillar is greyish-black, with light brown hairs and two reddish-brown lines on the back, and has a black protuberance on the fifth and last segments.

### OTHER BRITISH VENOMOUS CATERPILLARS.

There are four other British caterpillars which deserve notice on account of their urticating properties. The larva of the drinker moth is a dark brown striped with yellow on the sides, and it has a black tuft of hair at each extremity. Its hairs, though short, are very irritating. The lappet caterpillar (for it and not the moth ought to be so designated) is ashy-grey or light brown. On the back of the neck are two blue marks, and along the sides a series of fleshy protuberances—the so-called lappets. The caterpillar of the oak-egger, if the body is straight, appears of a uniform brown, but, if curved, velvet-black appears between the segments. There are white spots on the back, and a yellow mark on the head. The larva of the fox moth is black, with rather long golden hair.

Schoolboys with a taste for entomology are apt to touch caterpillars and get a rash which may appear at first sight like measles. It is when these hairy caterpillars are about to moult that they are most irritating, the hairs being then more rigid and brittle.

### FOREIGN VARIETIES.

Some foreign caterpillars are exceedingly venomous. In France some of the alleys of the Bois de Boulogne were closed to the public in 1865 because of the annoyance caused by the caterpillars of the processionary moths. The French newspapers teem with notices of "poisonings" by these caterpillars. In 1862 a boy, near Lyons, was climbing a tree when he shook an immense number of them down upon him so that they fell inside his shirt. His skin was covered with large red spots, and he died in a few hours with fever and delirium. The caterpillar of the sting moth in Australia caused a death, and a case of gangrene occurred in India from the bristles of the *shoa poká*, or the hairy caterpillar. Livingstone found that the bushmen of South Africa used the entrails of a small caterpillar called *n'gwa* as an arrow poison. If in putting it on the barb of an arrow a small portion got into a scratch the agony of the sufferer was terrible, and he sometimes went mad.

FIG. 3. [*Drawn by H. A. Knight.*
GOLD-TAIL MOTH AND LARVA
(Porthesia Auriflua).

The hairs of various kinds of caterpillars were analysed over fifty years ago by Wills, and he declared the irritating substance to be formic acid, but other investigators have since contended that it is allied to cantharidin.

## Figures and Tables

**Fig. 1. f1:**
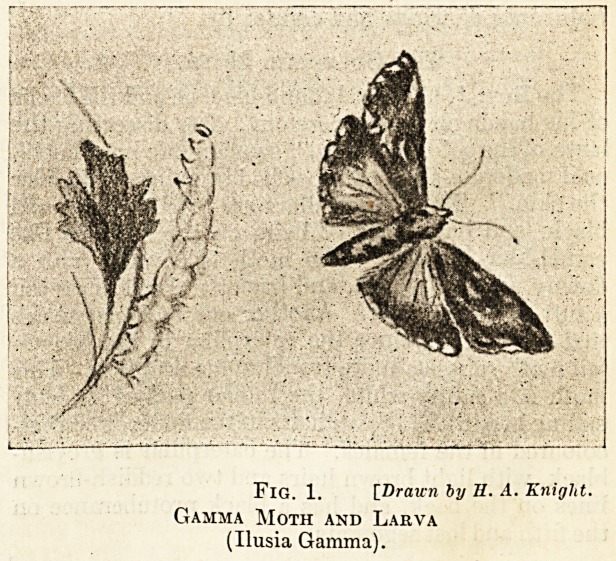


**Fig. 2. f2:**
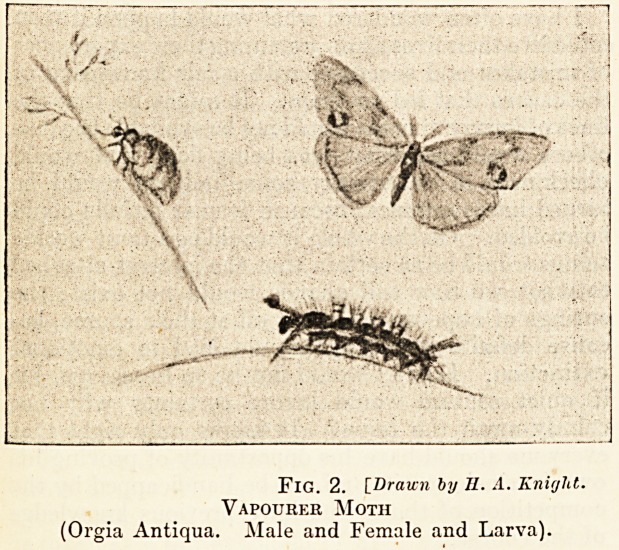


**Fig. 3. f3:**